# The effects of the prevention program ‘New Perspectives’ (NP) on juvenile delinquency and other life domains: study protocol for a randomized controlled trial

**DOI:** 10.1186/2050-7283-2-10

**Published:** 2014-04-16

**Authors:** Sanne LA de Vries, Machteld Hoeve, Jessica J Asscher, Geert Jan JM Stams

**Affiliations:** Research Institute Child Development and Education, University of Amsterdam, Nieuwe Prinsengracht 130, Amsterdam, 1018 VZ The Netherlands

**Keywords:** Effectiveness, Randomized controlled trial (RCT), Delinquency, Adolescents, Prevention, Care as usual

## Abstract

**Background:**

New Perspectives (NP) is a prevention program aiming to prevent that youth at onset of a criminal career will develop a persistent criminal behaviour pattern. The effects of NP on juvenile delinquency and other life domains are investigated, using a randomized controlled trial (RCT).

**Method/Design:**

In the present study at-risk youth aged 12 to 23 years are assigned randomly to the intervention (*N* = 90, NP) or control condition consisting of care as usual (*N* = 90, CAU). After screening, random assignment, and consent to participate, adolescents and their parents are requested to complete questionnaires. Data are collected at four points in time: at baseline (before the start of the intervention), after 3 months, after 6 months (post-test) and 1 year after treatment (follow-up). Primary outcome measures include involvement in delinquent behaviour and recidivism. Secondary outcome measures include parenting behaviour, life events, prosocial behaviour, deviant and prosocial peers, externalizing behaviour, cognitive distortions, moral reasoning, self-worth, anxiety, depression, client satisfaction, therapeutic alliance and motivation. Standardized questionnaires and interviews are used to collect data. Moderator analyses will also be conducted in order to examine the influence of ethnic background, gender and age on the program effectiveness.

**Discussion:**

The present study will provide new insights in the effects of a prevention program targeting youth at risk for the development of a persistent criminal career.

**Trial registration:**

Dutch trial register number NTR4370. The study is financially supported by a grant from ZonMw, the Dutch Organization for Health research and Development, grant number 157004006. The study is approved by the Ethics Committee of the University of Amsterdam, approval number 2011-CDE-01.

## Background

Juvenile delinquency can be considered as an important societal problem with negative consequences, such as mental health-, financial-, and work-related problems. Young offenders represent a relatively large proportion of all offenders in the justice system. For example, in 2003, juveniles in the United States accounted for 16% of all arrests (i.e., 2.3 million arrests), 15% of all violent crime arrests, 29% of all property crime arrests and 39% of all vandalism offences (Snyder & Sickmund 2006). The highest levels of prevalence rates of self-reported *total delinquency* (last year) among 12-15-year-old juveniles were found in cities of the United States, Ireland, the Netherlands and Germany (based on 43,968 respondents from 63 cities and 31 countries) (Enzmann et al. 2010). These countries also showed the highest rates of serious violent delinquency among youth. Approximately one third of the 12-to 17-year-old Dutch juveniles (38%) reported having committed a criminal offence (Van der Laan & Blom 2011).

Earlier studies showed that severe persistent delinquent behaviour of youngsters starts with minor offences and an accumulation of risk factors in multiple life domains, which could escalate in serious criminal offending (Loeber et al. 2009). In order to prevent that juvenile offenders will develop a chronic and persistent criminal career, there is a great urge for evidence-based prevention programs. Given the high costs of intensive treatment and incarceration of delinquents, investing in prevention could also contribute to economic benefits for society.

In the present study we will examine the effects of the prevention program ‘New Perspectives’ (NP), targeting juveniles at risk for the development of a persistent criminal career. This community-based program is acknowledged as a well implemented program with a strong theoretical foundation (Van den Braak & Konijn 2006). The NP program aims to prevent or reduce delinquent behaviour and offending. The theoretical framework of NP is based on the *Risk-Needs-Responsivity* (RNR) *model* (Andrews et al. 1990). Preventive and curative interventions are most likely to be effective when programs target criminogenic factors and are responsive to the individual needs of juveniles (Andrews & Dowden 2007). NP is also based on the *Transtheoretical Model of Behaviour Change* (Prochaska & Di Clemente 1984), which describes the stages of behaviour change in the context of treatment processes. Moreover, NP can be viewed as a multicomponent program addressing multiple risk factors by including multiple treatment modalities, such as elements of cognitive and problem-solving skills training and involvement of the social network (parents, peers and teachers, etc.). Multi-facetted programs integrating multiple components for parents, youths and their environment (school and community) are considered to be more beneficial than narrowly focused programs in juvenile crime prevention (McCord et al. 2001).

Previous evaluation studies of NP (Noorda & Veenbaas 1997; Geldorp et al. 2004) revealed positive results in various areas (such as school, family and peers) for NP youths. However, these evaluation studies lacked the use of a control group. Application of randomized controlled trial (RCT) provides the strongest evidence of causal relations between a participant’s exposure to treatment conditions and changes in deviant behaviour (Clingempeel & Henggeler 2002; Weisburd 2010). Therefore, the present study involves a randomized controlled trial.

On the basis of earlier international studies of programs aimed at preventing and reducing delinquency and recidivism, we expect to find evidence for positive effects of NP. Positive effects were found for diversion programs, stating that well-implemented programs, integrating behavioural and family-based change strategies, produced reductions in subsequent offences. These prevention programs targeted youth with only one or two police contacts, who have not yet exhibited a longstanding pattern of severe antisocial and delinquent behavior (Mulvey et al. 1993). Furthermore, a systematic review (Lösel & Beelmann 2003) indicated that well-structured multimodal cognitive-behavioural programs were most appropriate for preventing antisocial behaviour of adolescents. Hanlon and colleagues (2002) evaluated a multimodal and community-based prevention program, including individual counselling, mentoring and remedial education, targeting youths at risk for the development of a deviant lifestyle. This program proved to be effective in reducing delinquent activity in the long-term (1 year after the intervention). Thus, there is empirical evidence to suggest that multimodal prevention programs are effective.

However, in the international literature, there is no consensus on the degree of effectiveness of programs in preventing persistent delinquency. For example, a meta-analytic study (Deković et al. 2011) examined the long-term effects of prevention programs carried out during early and middle childhood on criminal offending into adulthood. They found no convincing evidence that early prevention programs are able to prevent adult crime. Most of the evaluation studies have focused on prevention in early or middle childhood (e.g. Deković et al. 2011) and on serious and chronic offenders (e.g. Asscher et al. 2007), but in the present study we will investigate the effects of a prevention program targeting youngsters at onset of their criminal career.

The program effectiveness of NP is examined in terms of decreased *delinquent behaviour* and *improvements* in life domains of juveniles, such as school, peers, and parents. Moreover, the study is focused on outcomes that are not directly addressed by NP, but are considered as factors related to delinquent behaviour, such as parental monitoring (Crouter & Head 2002), cognitive distortions (Barringa et al. 2000), self-esteem (Donnellan et al. 2005), and moral reasoning (Stams et al. 2006; Van Vugt et al. 2011). Given that externalizing behaviour problems often co-occur with internalizing problems (Barker et al. 2010), we also examine program outcomes related to depression and anxiety. Another important question of present study is related to the intervention effects for specific subgroups of youngsters. The NP client population in Amsterdam is very diverse with respect to ethnic background, gender and age. NP is also divided in different modalities for younger (below 16 years; NP ‘Preventief’ and ‘NP Plus’) and older adolescents (from 16 years; NP). In this respect it is important to detect possible differential effects of NP for these subgroups. In social work research and practice, there is little consensus about the need for, and effectiveness of, ethnically, gender-and age-tailored treatment (Wilson et al. 2003; Zahn et al. 2009). Although research consistently demonstrates that female juvenile offending is associated with specific risk factors (i.e., different from those of male juvenile offending) (Hipwell & Loeber 2006), gender-non-specific programs were found to be equally effective in reducing recidivism for boys and girls (Zahn et al. 2009). Also, a large amount of studies revealed that migrant children are at increased risk of mental health problems and experience specific risks related to stress and feelings of alienation due to the migration process (Stevens & Vollebergh 2008). Despite these different risk factors, mainstream service programs were found to be equally effective for minority and white juvenile delinquents in the United States (Wilson et al. 2003). Moreover, it is well known that the extent and impact of risk factors changes with age. For instance, the influence of peers in the adolescent’s behaviour increases with age, while the impact of parental supervision decreases with age (Loeber et al. 2006; Van der Put et al. 2011). Consequently, well-founded empirical knowledge about differential effects of prevention programs for different subgroups is needed.

Moreover, we are interested in the contribution of client factors (e.g., motivation, client satisfaction), client’s expectations and non-specific treatment factors to the program effects of NP. For example, the therapeutic alliance is assumed to have a strong impact on program outcomes (Karver et al. 2006). Also several researchers have indicated that the level of client satisfaction is related to behaviour improvements (Donovan et al. 2002). However, the unique contribution of these factors to treatment success remains unclear. The interrelation of clients’ expectations, therapeutic alliance, and specific treatment method is assumed to be complex. For example, therapeutic alliance can be promoted by professional appliance of specific methodical techniques (Stams et al. 2005) and client type and severity of psychopathology have been found to be associated with client satisfaction (Nock & Kazdin 2001).

There are, in particular outside the USA, relatively few randomized experiments in the field of criminology (Farrington & Welsh 2005). Experimental designs can rule out alternative explanations for program outcomes, such as passage of time, effects of assessment, or different types of clients (Cook 2003). By using an experimental design, the present study will be able to gain more insight into the effects of NP in preventing persistent delinquent behaviour and reoffending of at-risk youth. Our study focuses on youth at the onset of a criminal trajectory, who are at risk for persistent offending. This study will also provide more information about improvements in other life areas, such as relationships of youngsters with their parents and peers. In addition, moderators will be investigated in order to enhance the effectiveness of NP for divers target groups (young and older juveniles, boys and girls, different ethnic backgrounds). Finally, we will examine the contribution of non-specific treatment characteristics, client factors and client’s expectations to the intervention effects.

## Methods and design

### Aim of the study

The aim of this study is to examine the effectiveness of the prevention program ‘New Perspectives’ (NP) in a sample of youth at risk for the development and progression of a deviant life style. The effects of NP are compared with care as usual (CAU), the comprehensive interventions that are already available. We expect that NP will be more effective than CAU. The effectiveness will be measured in terms of decreased problem behaviour and improved quality of life. Primary outcomes are defined as a reduction in delinquent behaviour, offending, and recidivism. Furthermore, we will investigate improvements in the individual domain (e.g. self-esteem and cognitive distortions) and in life domains, such as school, peers, and parents. These factors are considered as mediators for the effectiveness of NP. The role of clients’ expectations (satisfaction), client factors (motivation) and non-specific treatment (treatment alliance) variables will be taken into account as well. Finally, potential moderators (age, ethnicity and gender) of the effectiveness of NP will be studied.

### Design

This study protocol will follow the CONSORT statement (Moher et al. 2010). The design of this study involves a randomized controlled clinical trial (RCT) in which NP will be compared to CAU. Data of adolescents and their parents will be collected at four points in time: prior to treatment (T1 pre-test assessment), after 3 months (T2 the intensive intervention phase), immediately after treatment (T3 post-test assessment, 6 months after T1, the aftercare phase), and 1 year after treatment (T4 follow-up 12 months after T3).

**Figure 1:**
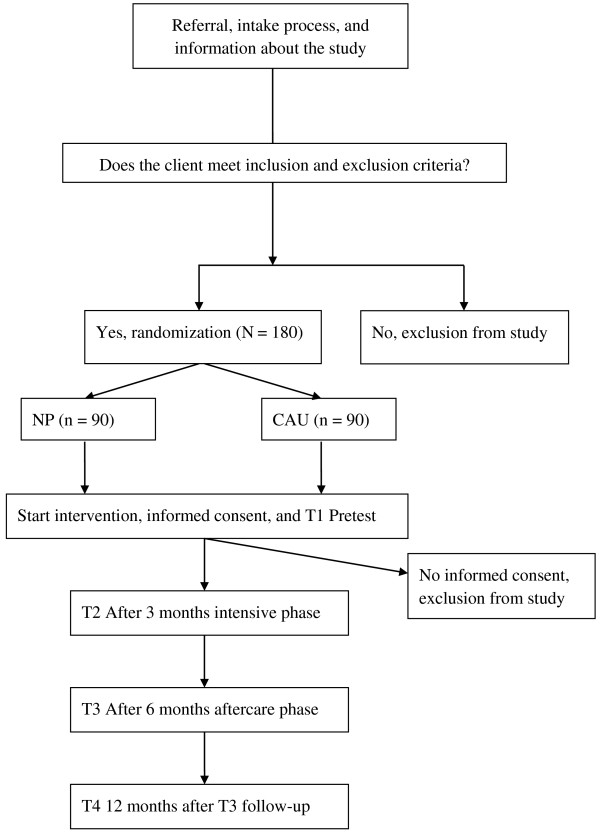
**Flow diagram NP effect study** ***.***

Adolescents aged 12 to 23, who meet the eligibility criteria of NP (these criteria are described in next section) will be randomly assigned to either NP or CAU. Random assignment per adolescent will be executed by the researcher (first author) using computer generated block randomization. The ratio of the randomization between NP and CAU is 1:1. See Figure 1 for the procedure’s flow chart.

The Ethics Committee of the University of Amsterdam (Faculty of Social and Behavioural Sciences) approved the study design, procedures and informed consent. Participation is voluntary and all participants (adolescents) will be asked to provide written informed consent at first assessment. Parental consent will be obtained when the adolescent is younger than 16.

### Sample size

Power calculations indicated that 90 adolescents per condition (assuming an alpha of 0.05, 0.95 power, and a medium effect size, based on power calculations of G*Power (Faul et al. 2009)), are sufficient to detect a difference in problem behaviour at post-test. There is also sufficient power to perform moderator-analyses for different subgroups (Power > .80 to detect small effects for 2 to 8 groups). Therefore, a total of 180 adolescents and parents will be included.

### Study sample

Adolescents are eligible for participation if they meet the following criteria: (1) age 12 to 23 years, (2) experiencing problems on multiple life domains (school, family, peers, leisure time), and (3) at risk for the development and progression of a deviant life style, such as predelinquents with antisocial behaviour, first time offenders and adolescents with mainly minor police contacts and offences (such as, purposely damage or destroy property, shop lifting and joyriding). Exclusion criteria are an IQ below 70, severe psychiatric problems, severe drugs-or alcohol use (dependency), absence of residence status in the Netherlands, and absence of motivation to stop committing criminal acts. NP-clients may be court-ordered, but are mainly referred by (primary or secondary) schools, social workers or they may be self-referred.

### Recruitment

The participants will be recruited via five locations of a large youth care institution in Amsterdam, the Netherlands. At the time of referral, adolescents and their parents will be informed about the NP-effectiveness study. After screening for the inclusion and exclusion criteria by clinical professionals at the youth care institution, adolescents are randomized to NP or to CAU. Immediately after randomization an appointment will be made in order to obtain written informed consent and to conduct the first assessment. The assessments will be carried out by junior researchers and master students (of Forensic Child and Youth Care Sciences). These students and researchers will be trained by means of a standardized protocol.

### Intervention

Youths in the experimental condition will receive the intervention New Perspectives (Elling & Melissen 2007), an intensive, short-term and community-based program targeting youth at risk for (persistent) juvenile delinquency. The main purpose of NP is to prevent or reduce delinquent behaviour and offending. Moreover, the program aims to improve the quality of life and addresses several key systems (home, school, peers and neighbourhood) in which the juvenile is embedded. The target group consists of at-risk youth from 12 to 23 years who are confronted with a sum of risk factors, in domains such as individual behaviour, family and friends, school/work, and neighbourhood. The NP program consists of an intensive coaching phase of 3 months followed by a 3-month aftercare phase. The total duration of the program is 24 weeks. Youth care workers, who have low caseloads, are available 24 hours a day, seven days per week. The average contact intensity per week is 8 hours per client. The following core activities and modalities are carried out by youth care workers: setting goals (in consultation with the client), coaching and confronting, motivational interviewing, empowerment and reinforcement of the social network (involvement of parents, peers, teachers, etc.), practical support, cognitive restructuring, problem-solving skills, and modelling (social workers act as role models) (Elling & Melissen 2007).

The control condition consists of care as usual (CAU), other existing standard services of youth care in Amsterdam. These services include child welfare services, such as family and/or individual counselling, social and/or cognitive behavioural skills training, academic service coaching, and mentoring.

### Data collection process

Adolescents and parents will complete self-report questionnaires using an online computer program at home. Both questionnaires have a login code to secure privacy. Youth will receive €20 and parents €10 per completed assessment. The youth care workers will fill out three questionnaires directly after the intensive intervention phase. The data will be treated as confidential: participants receive a unique code which is used for the online computer program and other research documents. Names are omitted and researchers declare that they will not provide any information of participants to third parties without their permission.

### Instruments

Table 1 shows the concepts, sources, and times of assessment for all used instruments. Most questionnaires will be administered at all measurement moments, except for the questionnaires of *intelligence*, *client satisfaction*, *motivation*, *therapeutic alliance* and *moral reasoning*. The questionnaires concerning treatment can only be filled out during the intervention phase (T2 en T3). The other two questionnaires (intelligence and moral reasoning) are filled out at one or two assessment moments in order to avoid overcharge of the respondents.

**Table 1:** **Instruments at different assessments and informants**

Outcomes	Concept	Instrument	Items	Source			Assessment			
				A^1^	P^2^	S^3^	T1	T2	T3	T4
Primary	Delinquency	SRD	33	x			x	x	x	x
	Recidivism	Officialª								
Secondary	School/work situation	Official								
	Parenting behaviour	PBQ	30	x	x		x	x	x	x
	Parental monitoring	VTH	6	x	x		x	x	x	x
	Family functioning	VGFO	28		x		x	x	x	x
	Life events	VGFO	15		x		x	x	x	x
	Parental attachment	IPPA	12	x			x	x	x	x
	Peer affiliations	FFS	17	x			x	x	x	x
	Contact intensity peers	BVL	5	x			x	x	x	x
	Prosocial behaviour	PBQ	20	x			x	x	x	x
	Externalizing behaviour	SEV	72		x		x	x	x	x
	Substance dependency	CRAFFT	6	x			x	x	x	x
	Aggressive behaviour	BDHI-D	35	x			x	x	x	x
	Depressive behaviour	CDI-2	27	x			x	x	x	x
	Anxiety	SCAS	45	x			x	x	x	x
	Cognitive distortions	HIT	54	x			x	x	x	x
	Self-esteem	CBSA	5	x			x	x	x	x
	Moral reasoning	SRM-SF	11	x			x		x	
	Intelligence	GIT	40	x						x
	Social desirability	SDS	15	x			x	x	x	x
Treatment	Client satisfaction	C-toets	22	x				x	x	
	Motivation	VMB	12	x		x	x	x	x	
	Therapeutic alliance	TASC	12	x		x		x	x	
	Program integrity					x		x		
Moderators	Demographic factors	Gender	1	x	x		x			
		Ethnicity	1	x	x		x			
		Education	1	x	x		x			
		Income	1		x		x			
		Living	1	x			x			

^1^A = adolescent; ^2^P = parent; ^3^S = social worker; ªRecidivism: Official reports about arrests and reoffending of Policy Database for Judicial Documentation; School/work: Official reports of local government (DMO and DWI) about registration, truancy and drop-out.

### Primary outcome measures

The primary outcome measure is the presence of *delinquent behaviour* among adolescents*.* Participation, frequency and versatility in offending, will be assessed by the ‘Self-report Delinquency Scale’ (SRD) (Van der Laan et al. 2009; Van der Laan & Blom 2006). The SRD scale consists of 33 items divided in three types of delinquent behaviour: *violent crime*, *vandalism*, and *property crime*. The acts range in severity from vandalism and petty theft up to injuring someone with a knife or other weapon. First, for the 33 types of offending activities, participants will be asked if they had ever been involved in each of these acts. Examples of items are: “Have you ever wounded anyone with a knife or other weapon?” and “Have you ever covered walls, buses, or entryways with graffiti?” Next, for each of the acts, where respondents answer with “yes”, they are then asked how often they participated in diverse delinquent acts during the past 3 months. *Recidivism* will be assessed with data of the Research and Policy Database for Judicial Documentation. This database provides information on the number of arrests, type and severity of offence of adolescent’s reoffending during the research period.

### Secondary outcome measures

The present study is based on a broad range of secondary outcome measures. Information about the *school* and *work situation* will be assessed by using the database of the local government in Amsterdam (*Dienst Maatschappelijke Ontwikkeling*, DMO and *Dienst Werk en Inkomen*, DWI). These data provide information about registration, drop-out rates, and truancy.

Parenting Behaviour, in particular *warmth*, *responsiveness* (parental support), *explaining*, *autonomy* (authoritative control), *strictness* and *discipline* (restrictive control), will be assessed with the ‘Parenting Behaviour Questionnaire’ (PBQ) (Wissink et al. 2006). The PBQ is applicable for different ethnic groups and could be used for both parental and juvenile reports. *Parental monitoring* will be measured by the ‘Vragenlijst Toezicht Houden’ (VTH), the Dutch version of the parental monitoring scale of Brown and colleagues (1993). Adolescents fill out how much their parents know about who their friends are; how they spent their money; where they were after school; which place they went when they left home; what they did in their leisure time; and what grades they received at school. Family Functioning will be assessed by the ‘Vragenlijst Gezinsfunctioneren Ouders’ (Janssen & Veerman 2005) based on five scales: *basic care*, *parenting*, *social contacts*, *childhood experience*, and *partner relation. Life Events* of the family will be measured by the ‘Vragenlijst Meegemaakte Gebeurtenissen’ (VMG) (Veerman et al. 2003). This questionnaire is based on parental reports about 15 specific life events. Parents fill out the specific period of the life event and whether the life event was experienced *positive* or *negative* by their child. The *quality of parent-adolescent relationship* will be assessed by using the short Dutch validated version of the ‘Inventory of Parent and Peer Attachments’ (IPPA) (Buist et al. 2004; Gullone & Robinson 2005). This instrument is designed to assess the extent to which adolescents felt secure by measuring the adolescents’ *trust* in availability and sensitivity of the attachment figure, the quality of *communication* and the extent of *anger and alienation* in the relationship with the attachment figure.

Adolescents’ perceptions of peer affiliation will be measured by the Dutch version of the ‘Friends’ scale which is a part of the ‘Family, Friends & Self Scale’ (FFS) (Deković et al. 2004; Simpson & McBride 1992). Adolescents indicate how many of their friends participated in a variety of deviant behaviours (e.g., purposely damage or destroy property). Affiliation with prosocial peers is measured by items of the FFS concerning prosocial activities (e.g. *good grades* and *sport*). The intensity of contact with peers is measured by a subscale of the ‘Basic Peer Questionnaire’ (BVL) (Weerman & Smeenk 2005). Adolescents answer how often they spend time with their peers during the week and weekends.

*Prosocial behaviour* of adolescents will be assessed by the ‘Prosocial Behaviour Questionnaire’ (PBQ) (Weir & Duveen 1981). This questionnaire is designed to measure positive aspects of adolescent’s behaviour. *Aggressive behaviour* will be measured by the Dutch self-report validated version of the ‘Buss-Durkee Hostility Inventory’ (BDHI-D) (Lange et al. 1994). The BDHI (Buss & Durkee 1957) consists of two subscales ‘Overt Aggression’ (measuring the tendency to express verbal or physical aggression) and ‘Covert Aggression’ (determining the emotional and cognitive components: hostility, irritability, suspicion, and anger). *Externalizing Behaviour* will be measured by the ‘Sociaal-Emotionele Vragenlijst’ (Social Emotional Questionnaire, SEV) (Scholte & van der Ploeg 2007). The SEV is based on the core symptoms of behaviour problems classified in the DSM and ICD: *attention deficits* and *hyperactivity*, *oppositional defiant*, *conduct* and *aggressive behaviour*, *anxiety*, *depression*, and *autistic behaviour*. Parents report how often their child shows problem behaviour. *Substance abuse* and *dependency* of adolescents will be measured by the CRAFFT Substance Abuse Screening Test (Knight et al. 2002). The CRAFFT is a specialized self-report screen to address both alcohol and drug dependency (Winters & Kaminer 2008).

*Internalizing problems* will be measured by the ‘Child Depression Inventory-2’ (CDI-2) (Breat & Timbremont 2002) and the ‘Spence Children’s Anxiety Scale’ (SCAS) (Spence 1998). The CDI-2 is a revision of the CDI (Kovacs 1985) and was translated in Dutch. This questionnaire is designed for measuring *depressive symptoms* (based on DSM-IV) of adolescents in different settings (at school; in child youth care settings). Adolescents report how they felt in the last two weeks. The SCAS is based on the DSM-IV and measures following symptoms of anxiety: *generalized anxiety*, *separation anxiety*, *social phobia*, *panic disorder*, *agoraphobia*, *obsessive-compulsive disorder* , and *specific phobia* (Spence 1998; Scholing et al. 1999).

*Cognitive Distortions* of adolescents will be assessed using the Dutch validated version of the ‘How I Think Questionnaire’ (Dutch version: HID) (Gibbs et al. 2001; Nas et al. 2005). The HIT is based upon four-category typology of self-serving cognitive distortions: *self-centred attitude*, *blaming others*, *minimizing-mislabelling* (consequences of) *behaviour*, and *assuming the worst* (Barringa et al. 2000). *Self-esteem* or feelings of worth and satisfaction with self will be measured by using the ‘Competentie Belevingsschaal voor Adolescenten’ (CBSA) (Treffers et al. 2002). This questionnaire is a Dutch version of the global self-worth subscale from the ‘Self-Perception Profile for Adolescents’ (Harter 1982). *Sociomoral Reasoning* of adolescents will be assessed by the ‘Sociomoral Reflection Measure–Short Form’ (SRM-SF) (Basinger et al. 1995). The SRM-SF addresses sociomoral values about *contract and truth*, *affiliation*, *life*, *property* and *law*. Adolescents are asked to evaluate and justify the importance of each value. The justificatory answers are scored for stages of moral reasoning (based on Kohlberg’s immature-mature stages). *Social Desirability* will be measured by the ‘Marlowe-Crowne Social Desirability Scale’ (SDS) (Crowne & Marlowe 1960). The SDS assesses the tendency of respondents to give socially desirable answers. *Intelligence* of adolescents will be measured by the ‘Groninger Intelligentie Test 2’ (GIT-2) (Verhage 1965). Three subtests of the GIT-2 will be used to indicate the level of intelligence of adolescents, namely *reasoning/induction and deduction* (‘Matrijzen’, 20 items), *visualization* (‘Legkaarten’, 20 items), and *numbers* (‘Cijferen’).

*Satisfaction* with treatment will be measured with the ‘C-toets’ (Jurrius et al. 2007), which has been designed for evaluating the satisfaction about treatment results of adolescents and their parents. *Motivation* for treatment of adolescents will be measured by the ‘Vragenlijst Motivatie voor Behandeling’ (VMB) (Van Binsbergen 2003). This questionnaire is based on the *Stages of Change Theory* (Prochaska et al. 1992) and presents the process of behavioural change in different stages. The *Therapeutic Relationship* will be measured by the ‘Therapeutic Alliance Scales for Children’ (TASC) (Shirk & Saiz 1992). The TASC is based on dimensions of (1) the child’s affective experience of treatment and (2) the child’s collaboration with the tasks of treatment. There is a client- and therapist version of the TASC. *Treatment Integrity* will be assessed by process evaluations consisting of analyses of program documents and protocols, structured interviews with program directors and staff, and observations (site visits). Moreover, we will conduct assessments with clinic personnel (social workers) through a structured program evaluation checklist which is based on the core elements of the intervention.

### Potential moderators

Information on demographic characteristics will be collected by adding questions about gender, ethnicity, age, education level, family income and situation of living to the self-report questionnaires.

### Statistical analysis

Primary analyses will be performed according to the intention-to-treat principle (Montori & Guyatt 2001). The effect of the intervention with regard to the difference in official arrest rates (recidivism) between the experimental and control group will be examined using logistic regression analysis and survival analysis. The primary (involvement in delinquency, SRD) and secondary continuous measures will be analyzed with ANCOVA using the outcome measures at post-test and follow-up as dependent variables, treatment condition as factor and pre-test scores as covariates.

Moderator analyses will be conducted using two-way ANCOVA’s with the moderators and treatment condition as factors, to examine interaction effects. For each questionnaire, the effect size is computed as Cohen’s *d*, based on adjusted means and standard errors, with a positive sign indicating improvement in the NP group relative to the control group. Mediator effects will be analyzed using structural equation modelling.

## Discussion

This article describes the study protocol of a program evaluation of the prevention program ‘New Perspectives’ (NP). This study is one of the few randomized clinical trials in Europe examining a program targeting youth at risk for the development of a persistent criminal career (Farrington & Welsh 2005). By conducting an experimental research strategy (RCT) we will be able to control for confounding effects more accurately than in studies with other designs. Furthermore, there are several strengths with regard to the design of the present study.

First, this evaluation study is carried out in the routine youth care practice, which contributes to the ecological validity of the findings. In addition, the use of an active control condition (care as usual) under real life conditions gives more insight in the unique contribution of NP compared to standard youth care interventions. This information is crucial for practitioners, policy makers and politicians in order to determine which prevention programs can best be implemented.

A second strength is the examination of potential moderators and mediators. We focus on moderators, such as ethnicity, age and gender. Moderator analyses establish under which circumstances interventions are effective in reducing problem behaviour (Clingempeel & Henggeler 2002). Through this method we could detect whether NP is effective with older or younger adolescents, boys or girls, and with adolescents from different ethnic backgrounds. Further, our study includes diverse secondary outcome measures (e.g., cognitive distortions) leading to a better understanding of processes that could mediate the relation between the intervention and delinquent behaviour.

Third, when examining the effects in terms of delinquent behaviour we distinguish between involvement in, frequency and seriousness of delinquent acts. These specific measures of criminal offending contribute to a more detailed view on program effectiveness (Farrington & Welsh 2005). Moreover, the investigation of long-term effects up to one year after the intervention could identify possible sleeper effects.

Finally, the role of general treatment factors, such as the therapeutic alliance, are also taken into account. This will lead to a better understanding of the influence of non-specific treatment factors on the program effects, and the unique effects of specific treatment factors over non-specific treatment factors.

Despite these strengths several pitfalls of this study design should be mentioned. One of the greatest challenges in conducting randomized experiments is avoiding drop-outs of respondents. In order to decrease the risk of drop-outs, we will apply a pre-randomization trial. The randomization will be conducted before active informed consent of respondents, which promotes random allocation and improves inclusion of participants. As a consequence, we need full cooperation of all referral institutions in providing sufficient information about the effect study before randomization. Therefore, we will actively inform all referral institutions in Amsterdam about the research design. In order to gain full cooperation of all institutions, we will start informing management staff of the most important youth care organizations in Amsterdam. Next, all involved institutions will receive detailed instructions about the study design through presentations of the researchers (on local levels).

Furthermore, in order to avoid drop-out during the research period, we will minimize efforts of youths and their parents through the application of online questionnaires. Researchers will visit respondents in their own environment (at school, at home, etc.). The youth care workers will facilitate the assessments by inviting researchers directly after their client appointments. At first assessment, youths and parents will be clearly informed about the importance and content of the study.

A final important risk of the present study design concerns the use of an active control condition (care as usual). Comparing NP to an active control condition (of other standard interventions) may lead to an underestimation of the mean effect size. The heterogeneous nature of the CAU condition and the possible evidence-based treatments (e.g., CBT) within this condition could result in a lower mean effect size. This methodological problem will be reduced by increasing the power.

## Conclusion

The present study will provide more insight in the effects of the prevention program ‘New Perspectives’ (NP) on a broad range of outcomes. More specific knowledge will be obtained about potential mediators of the effectiveness of NP, the role of non-specific treatment factors and the effects for different subgroups of youngsters. This information will contribute to improvement of programs for juveniles at risk for the development of a persistent criminal career.
